# Microenvironment-defined priority pathways shape ICD-driven abscopal responses

**DOI:** 10.3389/fimmu.2026.1933591

**Published:** 2026-08-13

**Authors:** Xiaobo Sun, Dandan Guo, Zhenrong Wang, Peixin Sun

**Affiliations:** 1 Department of Neurosurgery, Cancer Hospital of China Medical University, Liaoning Cancer Hospital & Institute, Cancer Hospital of Dalian University of Technology, Shenyang, Liaoning, China; 2 School of Biomedical Engineering and Technology Innovation, Fudan University, Shanghai, China; 3 Department of Thoracic Surgery, Cancer Hospital of China Medical University, Liaoning Cancer Hospital & Institute, Cancer Hospital of Dalian University of Technology, Shenyang, Liaoning, China

**Keywords:** abscopal effect, ICD, immunology, TME, tumor heterogeneity

## Abstract

While radiotherapy excels at local tumor control, it seldom orchestrates durable systemic immunity in the context of metastatic disease. The abscopal effect, the regression of non-irradiated distant lesions following focal irradiation, remains a clinical rarity rather than a predictable or robust consequence of monotherapeutic radiotherapy. Clinical manifestations of this phenomenon predominantly emerge within the framework of combinatorial radio-immunotherapy, specifically alongside immune checkpoint blockade, rather than as a byproduct of radiotherapy in isolation. This clinical reality underscores that radiotherapy-induced immunogenic cell death (ICD), though mechanistically pivotal, is rarely self-sufficient in catalyzing systemic antitumor immunity. We contend that the variability of the abscopal response cannot be ascribed solely to the presence or magnitude of radiotherapy-induced ICD; rather, it hinges decisively on how these ICD signals are “interpreted” across heterogeneous tumor microenvironments (TMEs). We propose a “priority pathway”: a functionally dominant, TME-dictated axis of ICD signaling that orchestrates immune cell polarization, infiltration dynamics, and the overall trajectory of the antitumor response. Under this paradigm, uniform ICD signals, encompassing calreticulin exposure, ATP release, HMGB1 signaling, and cGAS-STING activation, yield profoundly divergent outcomes, contingent upon stromal architecture, myeloid landscapes, vascular integrity, and metabolic constraints. We present testable predictions and delineate the dynamic, temporal shifts in post-irradiation pathway dominance, offering a mechanistic rationale for the heterogeneity and transience of the abscopal effect. We synthesize these insights into an investigational precision strategy designed to remodel the TME and augment ICD signaling in a phased manner, thereby breaching immunosuppressive barriers, potentiating T-cell-mediated systemic immunity, and enhancing the reproducibility of abscopal responses in radio-immunotherapy. These propositions are intended to catalyze future preclinical and clinical inquiry, serving as a hypothesis-generating framework rather than a set of established clinical doctrines.

## 1 Introduction

The abscopal effect was first documented over six decades ago, when radiotherapy directed at a single tumor site was observed to induce regression of distant, non-irradiated lesions. Despite the mechanistic interest this phenomenon has generated, it remains an exceptionally rare and poorly reproducible clinical event. Radiotherapy has been used widely in patients with advanced cancer for many decades, yet clinically evident regression of distant, non-irradiated lesions attributable to radiotherapy alone has never moved beyond isolated anecdotal observations. Pre-existing immune tolerance represents the central barrier restricting the development of effective systemic antitumor immunity.

The 2012 report by Postow et al. constitutes an important conceptual milestone in this field. The authors described a patient with metastatic melanoma receiving ipilimumab who experienced regression of non-irradiated lesions following palliative radiotherapy (1). The significance of this report lies not in demonstrating that radiotherapy alone can reproducibly generate abscopal responses, but rather in suggesting that radiotherapy may augment systemic antitumor immunity within the context of immune checkpoint blockade. This was a single-patient observation and should not be interpreted as evidence that abscopal effects can be reliably achieved in routine clinical practice. Nonetheless, the case and the mechanistic inquiry it stimulated together support the view that clinical discussion of the abscopal effect should be situated primarily within the framework of combined radio-immunotherapy, rather than treated as an attainable goal of radiotherapy used in isolation.

These observations have been tested prospectively. PEMBRO-RT and its pooled MDACC analysis showed that SBRT added to pembrolizumab improved outcomes in advanced NSCLC, most notably in PD-L1-negative patients, suggesting radiotherapy may compensate for low baseline immunogenicity only within a combination context (2, 3). A randomized trial in head and neck cancer found no significant benefit from adding SBRT to nivolumab in unselected patients (4). Formenti et al. demonstrated abscopal responses with radiotherapy plus CTLA-4 blockade alongside ICD-consistent immunological correlates (5). These data collectively support the view that radiotherapy alone rarely generates systemic responses, and that combination with checkpoint blockade is the meaningful clinical framework.

The immune basis of the abscopal effect has been reasonably well characterized in preclinical models. In bilateral syngeneic mouse tumor models, unilateral radiotherapy combined with FLT3L suppressed the growth of both irradiated and non-irradiated tumors in a strictly T cell-dependent manner. CD8^+^ T cell activation and infiltration into distant lesions are essential requirements in these experimental settings, as their depletion completely abolishes the effect (6, 7); systemic expansion and trafficking of tumor-specific T cells are correspondingly associated with abscopal responses (8). In the clinical setting, the inability to generate or recruit sufficient tumor-specific T cells remains the principal reason abscopal responses occur so infrequently (9).

**Figure 1:**
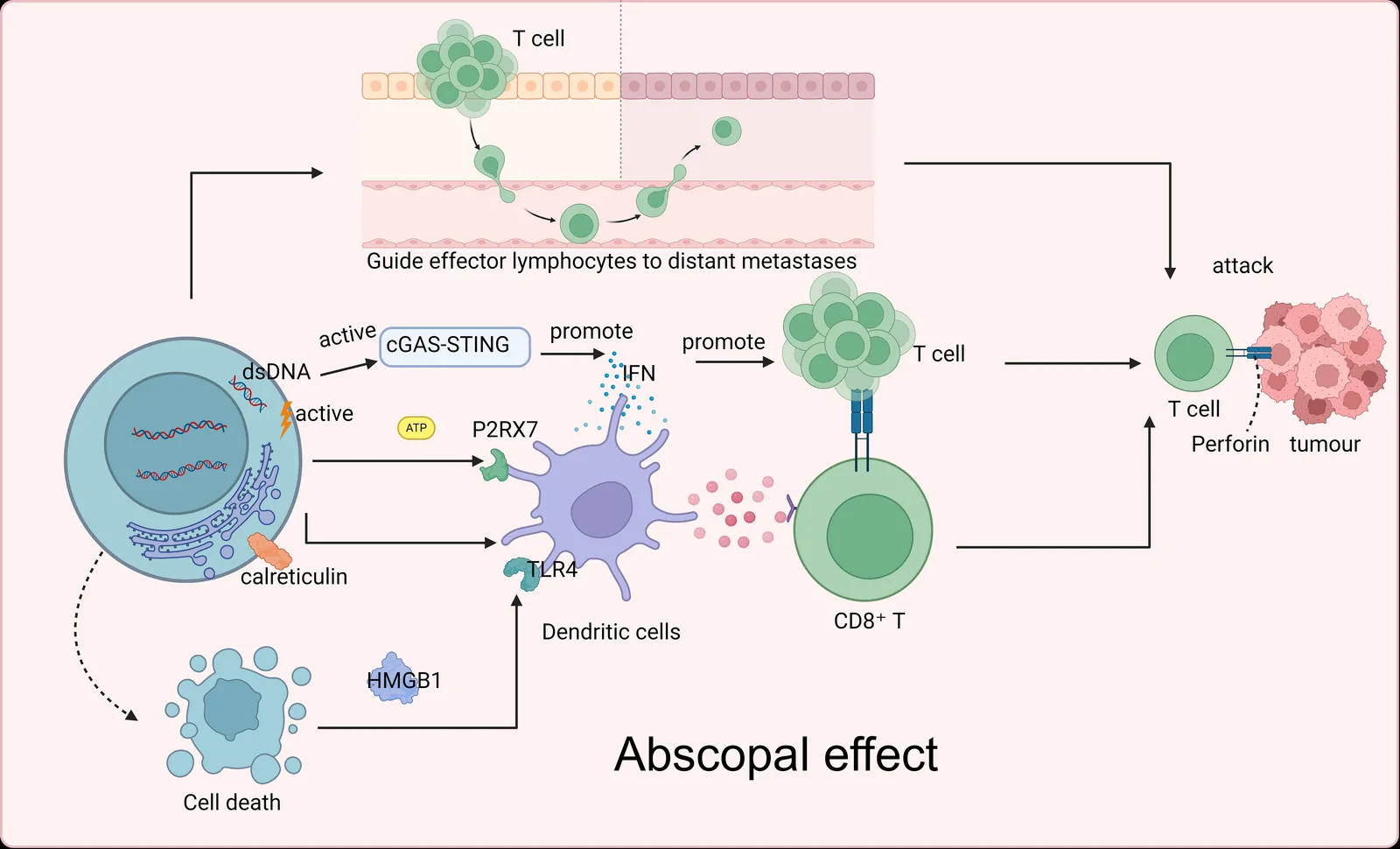
Preclinical mechanism of the abscopal effect.

At the mechanistic level, the available evidence derives predominantly from preclinical and *in vitro* studies. On the basis of this experimental evidence, radiotherapy-induced immunogenic cell death (ICD) has been proposed as the central driver of the abscopal effect. Unlike passive necrosis, ICD is a regulated cell death process with inherent immunostimulatory properties, in which dendritic cells serve as key intermediaries linking local tumor destruction to systemic T cell activation. The molecular hallmarks of ICD, as characterized in preclinical and *in vitro* systems, include calreticulin translocation to the cell surface as a phagocytic signal (10), autophagy-dependent ATP release activating dendritic cells through P2RX7 (11), HMGB1 binding to TLR4 to facilitate antigen processing (12), and cGAS–STING pathway activation driving type I interferon production to support dendritic cell maturation and T cell expansion (13). Importantly, excessively high radiation doses may upregulate TREX1, a cytosolic DNA exonuclease that degrades immunostimulatory DNA and suppresses STING activation, paradoxically attenuating rather than amplifying immunogenicity. In preclinical models, activated dendritic cells subsequently cross-present tumor antigens via MHC-I to CD8^+^ T cells, which expand into cytotoxic lymphocytes capable of circulating systemically and infiltrating distant tumor deposits (14, 15) (**Figure 1**). Whether this mechanistic cascade operates with comparable fidelity in human tumors, and whether it can be translated into clinically reproducible abscopal responses, remains an open question requiring dedicated prospective investigation.

The immunogenic potential of tumors and their responsiveness to radio-immunotherapy vary substantially across histological subtypes, a heterogeneity that must be explicitly acknowledged when designing and interpreting combination strategies. Melanoma and non-small cell lung cancer (NSCLC) occupy the most immunogenically permissive end of this spectrum: both harbor relatively high somatic mutational burdens generating abundant neoantigens capable of priming robust T cell responses (16, 17), and the majority of documented clinical abscopal responses have occurred in melanoma (1, 18). In NSCLC, the PACIFIC trial established that consolidation durvalumab following concurrent chemoradiotherapy conferred durable survival benefits (19), providing the strongest prospective clinical evidence that radiation-primed systemic immune activation can translate into meaningful outcomes. Mismatch repair-deficient (dMMR)/microsatellite instability-high (MSI-H) colorectal cancer similarly demonstrates substantial baseline immunogenicity and responds favorably to checkpoint blockade (20); however, the microsatellite-stable (MSS) majority, comprising approximately 85% of metastatic colorectal cancer, is characterized by a TGF-β-dominated immunosuppressive microenvironment that renders ICD-based strategies largely ineffective (21). Pancreatic ductal adenocarcinoma (PDAC) represents perhaps the most formidable barrier to radio-immunotherapy: its densely desmoplastic stroma physically excludes effector T cells (22, 23), while abundant immunosuppressive myeloid populations actively suppress antigen presentation and T cell function (24). Glioblastoma presents an additional layer of complexity through blood-brain barrier restriction of systemic T cell trafficking and IDO-mediated local immunosuppression (25). Intermediate histologies, including head and neck squamous cell carcinoma, urothelial carcinoma, and renal cell carcinoma, have demonstrated variable but encouraging responses to immune checkpoint blockade (26–28), and may represent underexplored candidates for precision radio-immunotherapy. These differential immunogenic profiles collectively underscore the necessity of histology-stratified trial design and microenvironment-informed patient selection in future radio-immunotherapy investigations.

This review draws predominantly on preclinical and translational evidence to position ICD as the proposed central driver of the abscopal effect in the context of combined radio-immunotherapy. We further argue that the TME may play a decisive role in determining whether ICD signals ultimately produce systemic immune activation or are redirected toward tolerance. We propose that two fundamentally distinct immunosuppressive architectures, the stroma-enriched and the myeloid-enriched microenvironment, may represent key candidate variables governing this outcome. On this basis, we introduce a “precision barrier-breaking” strategy that integrates functional TME subtyping with temporally coordinated interventions to amplify ICD-driven immunity while systematically suppressing competing inhibitory signals. This framework is a conceptual and investigational proposal rather than a clinically validated protocol, and is intended to provide mechanistic rationale and directional guidance for future preclinical and clinical research.

### 1.1 Common initiation points of ICD

Radiation-induced abscopal effects have been mechanistically attributed to ICD in preclinical models, and numerous physical and chemical treatment modalities share this capacity. Mapping the common upstream pathways of ICD may help interpret ICD-related signals across different treatment contexts. Across multiple ICD-inducing stimuli, the earliest consistently observed upstream events are PERK-mediated ER stress and eIF2α phosphorylation. Once eIF2α is phosphorylated, calreticulin translocates from the ER to the cell surface as ecto-CRT, which signals dendritic cells to engulf the dying cell. Anthracyclines, immunogenic doses of radiation, and hypericin-based PDT operate through distinct mechanisms yet all converge on PERK activation in experimental models, suggesting that ER stress induction is a required upstream step rather than an incidental consequence of cell death (29–33). ROS generation and mitochondrial dysfunction represent a second tier of conserved initiating events, acting upstream of or in parallel with ER stress. Radiotherapy generates ROS through water radiolysis, PDT produces singlet oxygen via photosensitizers, anthracyclines disrupt the mitochondrial electron transport chain, and dihydroartemisinin derivatives exploit tumor iron dysregulation through Fenton-like chemistry. Despite these different origins, the downstream outcomes converge in experimental models: mitochondrial membrane potential collapse disrupts calcium homeostasis and amplifies ER stress, while oxidative membrane damage promotes DAMP release. This suggests that mitochondrial dysfunction serves as a key amplification node in ICD pathways (34). The effector phase of ICD is defined by the sequential release of three core DAMPs: ecto-CRT externalization, followed by active ATP secretion, and then HMGB1 translocation from the nucleus to the extracellular space. This sequence carries clear functional significance. Ecto-CRT binds LRP1 on dendritic cells to promote phagocytosis and antigen cross-presentation; extracellular ATP activates P2RX7 receptors to trigger NLRP3 inflammasome assembly and IL-1β release; HMGB1 engages TLR4 to provide the maturation signal required for effective antigen presentation. The requirement for all three signals to appear in a coordinated temporal sequence explains why stimuli generating only a single DAMP often fail to induce authentic ICD (35).

Two additional mechanisms function as amplifying pathways in preclinical models. Cytoplasmic double-stranded DNA arising from radiation-induced micronuclei formation or mitochondrial DNA release activates the cGAS-STING/type I IFN axis, enhancing CD8^+^ T cell priming (36). Pyroptosis (mediated by gasdermin cleavage) and necroptosis (mediated by RIPK3/MLKL) release substantially more DAMPs than immunologically silent apoptosis due to plasma membrane disruption, and have been associated with stronger antitumor immune responses in preclinical models (37). Both pathways are supplementary amplifying mechanisms rather than core ICD requirements, and their relative contribution varies across tumor types and radiation regimens. Translating these findings into clinical practice requires acknowledging the technical limitations of directly measuring these molecular events in human tumor specimens. However, several clinically accessible surrogate markers are worth exploring as pharmacodynamic endpoints in translational studies. In tumor tissue, p-eIF2α and nuclear-to-cytoplasmic HMGB1 translocation can be assessed by immunohistochemistry in post-treatment biopsies using standard FFPE processing. In peripheral blood, circulating HMGB1 can be measured by ELISA, which is technically straightforward and has been explored after chemotherapy and radiation, though its specificity for ICD over other inflammatory processes remains unclear. Circulating cell-free mitochondrial DNA fragments may reflect cGAS-STING activation and are accessible through existing liquid biopsy platforms. Gasdermin D N-terminal fragments have been detected in plasma of patients with inflammatory conditions, suggesting potential utility as a pyroptosis biomarker after radiotherapy, though prospective validation in this setting is lacking. Before any of these approaches can meaningfully guide patient selection, standardization of assay conditions, sampling timepoints, and reference ranges remains an unresolved prerequisite.

Regarding patient stratification, tumors with high baseline immunogenicity, including those with high tumor mutational burden, microsatellite instability, pre-existing CD8^+^ T cell infiltration, or high STING expression, may be best positioned to convert radiation-induced ICD signals into meaningful antitumor responses, particularly when radiation is combined with immune checkpoint inhibitors. Immunologically cold tumors, characterized by sparse T cell infiltration and extensive immunosuppressive stroma, will likely require strategies targeting multiple barriers simultaneously before ICD signals can translate into effective immunity. The optimal dose, fractionation, irradiated volume, and timing of radiation relative to immunotherapy remain subjects of active investigation, and the framework presented here should be understood as a rationale for prospective trial design rather than a clinical decision guide.

**Figure 2:**
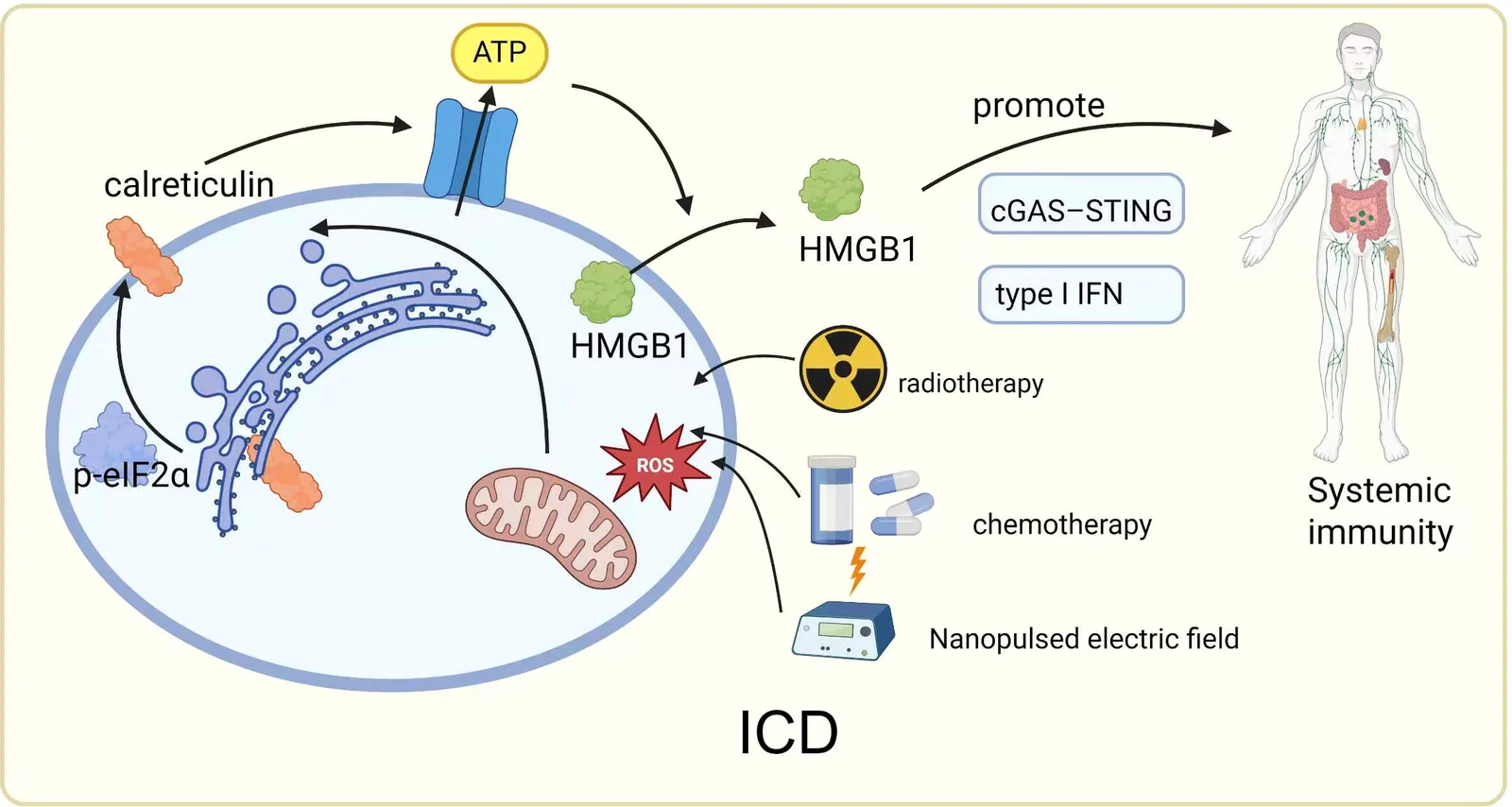
Molecular mechanisms of immunogenic cell death induction.

In summary, based on current preclinical evidence, the common ICD initiation sequence begins with PERK-mediated ER stress and eIF2α phosphorylation, is amplified by ROS-driven mitochondrial dysfunction, and culminates in the ordered DAMP cascade of ecto-CRT externalization, ATP secretion, and HMGB1 release, with cGAS-STING activation and inflammatory cell death serving as potentiating mechanisms. We propose that authentic ICD should be assessed by the temporally coordinated combination of these molecular events rather than any single marker, and that prospective translational studies incorporating on-treatment biopsies and pharmacodynamic biomarker co-endpoints are needed to determine whether this framework can guide patient selection and treatment sequencing in clinical practice (33, 35, 36, 38) (**Figure 2**).

## 2 Microenvironment-determined ICD priority pathway hypothesis

### 2.1 Definition of priority pathways and falsifiable predictions

Before analyzing microenvironment-specific ICD pathways, we define a “priority pathway” as an ICD-triggered signaling axis that meets three criteria: (i) dominant and sustained activation; (ii) functional control of immune cell polarization and infiltration; and (iii) restoration of antitumor immunity when inhibited. Priority pathway designation does not imply exclusivity: multiple ICD pathways may be active simultaneously, but only a subset controls immune outcomes under specific microenvironmental constraints (39, 40).

It is worth clarifying how the priority pathway framework relates to, and differs from, existing TME classification systems. The established immune-inflamed, immune-excluded, and immune-desert phenotypes classify tumors by immune cell presence, spatial distribution, and baseline inflammatory state. The priority pathway concept extends this framework by identifying the specific signaling axis through which ICD signals are processed in a given microenvironmental context. Two tumors classified as equally “immune-excluded” may be driven by fundamentally different dominant suppressive mechanisms: one through CAF-mediated physical and biochemical barriers and another through myeloid-dominated suppression. Conventional classification describes the phenotypic outcome but does not identify which upstream signaling axis drives it. Priority pathway designation attempts to identify this dominant functional axis to guide the choice of combination intervention most likely to redirect ICD signaling toward immune activation. This distinction may have therapeutic implications, but it currently remains a hypothesis requiring validation through biomarker-integrated prospective studies.

Pathway importance is not determined by the absolute amount of DAMP release or signal strength, but by whether the microenvironment allows that signal to shape immune outcomes. In stroma-enriched tumors, even when HMGB1 release is comparable to other tumor types, the HMGB1–TLR4 axis often fails to recruit dendritic cells effectively, because CAF-derived PAI-1 and SFRP2 create physical and biochemical barriers that block this process.

**Figure 3:**
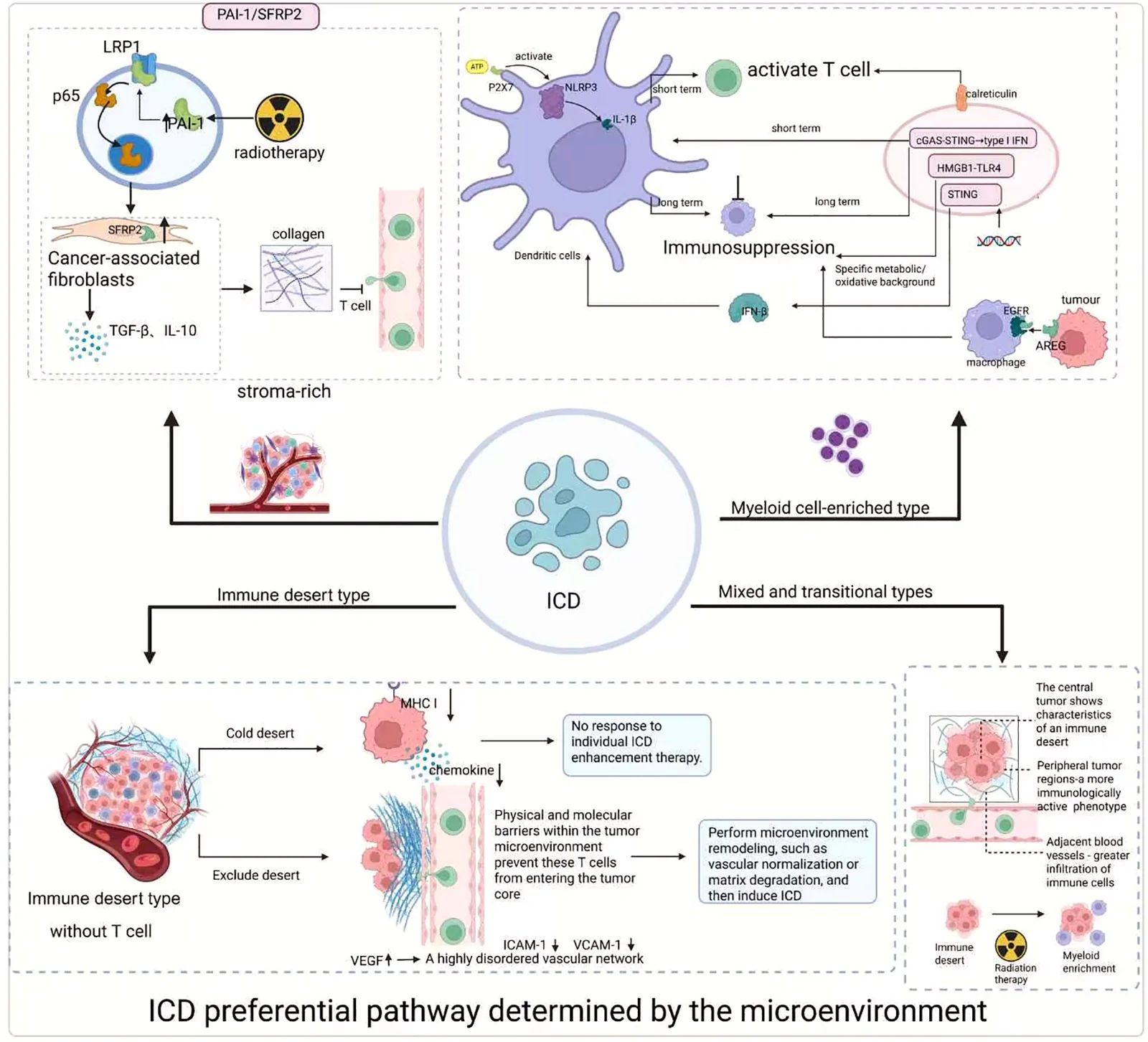
Conceptual model of the ICD priority pathway across five TME subtypes: stroma-enriched (PAI-1/SFRP2^high CAF axis blocks immune infiltration), myeloid-enriched (AREG–EGFR and ATP–P2X7 circuits redirect ICD toward tolerance), immune-desert (cold: absent T cell/DC infiltration; excluded: vascular dysfunction restricts T cell entry), and mixed/transitional states. Arrows indicate the dominant suppressive circuit per subtype. All designations are hypothesis-driven.

This model generates four testable hypotheses, presented here as hypothesis-generating predictions based on preclinical observations. First, disrupting the dominant pathway should shift the pathway hierarchy: in stroma-enriched tumors, CAF modulation via FAP-directed strategies or losartan should reduce PAI-1/SFRP2 suppression and allow HMGB1–TLR4 or ATP–P2X7 signaling to become more active, potentially increasing CD8^+^ T cell infiltration (41, 42). Second, targeting priority pathways should yield greater benefit than targeting non-priority ones: in myeloid-enriched tumors, AREG blockade should outperform stromal-targeting strategies. Third, metabolic context should influence pathway hierarchy: under normoxic, low-lactate conditions, classical ICD pathways may regain activity, and metabolic interventions should shift myeloid polarization toward pro-inflammatory states (43, 44). Fourth, priority pathways evolve over time after radiotherapy, with early responses driven by rapid ICD signals (calreticulin, ATP) and suppressive microenvironmental circuits emerging later (45). Standard TME classification uses immune cell counts but does not capture the functional interactions between ICD signaling and microenvironmental components. We propose a functional classification that incorporates cellular composition, vascular features, stromal organization, metabolic status, and dominant immunosuppressive circuits (46). This framework identifies five subtypes: stroma-enriched, myeloid-enriched, immune-desert, vascular-abnormal, and mixed/transitional states, each associated with distinct priority pathways (**Figure 3**; **Table 1**).

**Table 1:** Summary comparison of five TME subtypes within the priority pathway framework, detailing core biomarkers, targeted agents, RT parameters, and monitoring timepoints.

TME Subtype	Core Biomarkers	Recommended Targeted Agent	Optimal RT Parameters	Monitoring Timepoints
Stroma-enriched	SFRP2-high CAFs, PAI-1↑, α-SMA, collagen density	PAI-1 inhibitors, SFRP2-neutralizing antibodies, TGF-β blockade, PD-1/PD-L1 inhibitors	Moderate hypofractionation (6–8 Gy/fraction); avoid high single doses (>12 Gy) that maximally induce PAI-1	Baseline; Day 3–7 (PAI-1 in plasma); Week 3–4 (stromal remodeling by DW-MRI)
Myeloid-enriched	TAM/MDSC density, AREG↑, CD47↑, IDO, PD-L1	Anti-CD47/SIRPα, AREG inhibitors, CSF1R inhibitors, STING agonists (intratumoral)	Moderate hypofractionation (8–12 Gy/fraction); time immunotherapy to 48–72 h post-RT window	Baseline; Day 3–7 (AREG, exosomal DAMPs); Week 3–4 (myeloid composition, ctDNA)
Immune-desert (Cold)	MHC-I loss, absent CXCL9/10/11, low TIL density	STING agonists, TLR agonists, oncolytic viruses + checkpoint blockade	Ultra-low-dose fractions (0.5–1 Gy per fraction, cumulative 6–13 Gy over multiple cycles); avoid large-field ENI	Baseline molecular profiling; Week 3–4 (MHC-I re-expression, T cell trafficking)
Vascular-abnormal / Immune-desert(Excluded)	VEGF↑, ICAM-1/VCAM-1 loss on endothelium, high interstitial pressure, FasL+ endothelium	Low-dose anti-VEGF (vascular normalization), PEGPH20, losartan, CXCL10-inducing agents	RT dose per lesion-specific priority pathway combined with anti-VEGF-mediated vascular normalization in staged sequence; staged approach	Baseline vascular imaging; Day 3–7 (DW-MRI, CXCL10); Week 3–4 (T cell infiltration)
Mixed/Transitional	Spatial heterogeneity on multiplex IF, co-existing CAF and myeloid compartments	Staged combination: stromal disruption (Week 1) → myeloid targeting (Week 2–3) → checkpoint blockade	Regimen guided by dominant compartment at biopsy; serial adaptation required	Longitudinal serial biopsies or ctDNA + imaging at all three timepoints

All recommendations are hypothesis-driven and require prospective clinical validation.

The distribution of dominant priority pathways is not uniform across histologies. PDAC exemplifies the stroma-enriched subtype, in which dense desmoplasia renders the PAI-1/SFRP2 axis dominant, physically excluding effector T cells regardless of ICD signal strength (22, 23, 47). Melanoma more frequently presents as myeloid-enriched or immune-inflamed, with AREG–EGFR and HMGB1–TLR4 signaling competing as priority axes, such that myeloid polarization largely determines ICD outcome (1, 48). NSCLC spans mixed and myeloid-enriched subtypes, where cGAS–STING activation and TGF-β-driven exclusion co-dominate and may shift with radiation dose or prior therapy (17, 19, 42). Microsatellite-stable colorectal cancer is characterized by TGF-β-dominated myeloid enrichment in which AREG-mediated reprogramming suppresses antigen presentation despite adequate DAMP release (21). Glioblastoma predominantly occupies the immune-desert subtype, with IDO-mediated local suppression and blood-brain barrier restriction constituting co-dominant pathways (25). These associations reflect predominant microenvironmental features and are intended to guide hypothesis generation rather than prescribe fixed pathway assignments. These categories are not static: radiotherapy remodels the microenvironment, and longitudinal monitoring may therefore be necessary. The tools required for comprehensive functional TME subtyping, including spatial transcriptomics, multiplex immunofluorescence, and imaging mass cytometry, are currently available mainly in research settings and are not part of routine clinical practice, underscoring the investigational nature of this proposed classification (49, 50).

### 2.2 Stroma-enriched and myeloid-enriched microenvironments: divergent priority pathways

The mechanistic analysis in this section is based primarily on preclinical experimental evidence, with limited direct clinical validation. The proposed pathway hierarchies should be read as biologically plausible hypotheses, not clinically established mechanisms.

Radiotherapy parameters, including dose per fraction, irradiated volume, and timing relative to immunotherapy, have mechanistically distinct effects on ICD induction and should be considered when designing or interpreting radio-immunotherapy studies. The relationship between radiation dose and immune stimulation is non-linear.

These dose–response relationships carry distinct implications across TME subtypes. In stroma-enriched lesions, moderate hypofractionation is preferable to ablative doses: very high single fractions (>12–15 Gy) further amplify PAI-1 induction and accelerate SFRP2^high CAF transdifferentiation at both irradiated and distant sites (47), thereby reinforcing rather than dismantling the dominant suppressive architecture, while moderate doses retain sufficient cGAS–STING activation and DAMP release to initiate the ICD cascade (51). In myeloid-enriched lesions, the optimal range is 8–12 Gy per fraction: conventional fractionation (1.8–2 Gy) produces insufficient STING activation to overcome the suppressive myeloid threshold (52), whereas ablative doses (>12 Gy per fraction) upregulate TREX1 and suppress cGAS–STING-driven interferon production (53); furthermore, under the hypoxic, lactate-rich conditions typical of this subtype, sustained STING signaling paradoxically upregulates PD-L1 and IDO, converting immunogenic signals into a tolerogenic program (54). Myeloid-targeting agents such as AREG inhibitors or CSF1R blockade should be administered within 48–72 hours post-irradiation, before suppressive macrophage and MDSC programs peak (55). Elective nodal irradiation should be avoided in both subtypes to preserve circulating lymphocytes essential for systemic antitumor immunity (56).

ICD is a feature of many anticancer therapies. Under controlled experimental conditions, its hallmarks (calreticulin exposure, ATP release, HMGB1 secretion, and type I interferon induction) activate dendritic cells and promote T cell responses. In clinical settings, however, outcomes depend heavily on the TME. Stroma-enriched and myeloid-dominated tumors illustrate how the same ICD signals can produce different immune outcomes depending on which cellular components are present to process them (**Figure 3**).

In stroma-enriched tumors, radiotherapy elevates PAI-1, which triggers pericyte-to-SFRP2^high CAF transdifferentiation via LRP1/p65 signaling. These CAFs accumulate around vessels and block immune cell entry (47). In preclinical models, SFRP2 knockout in CAFs improves abscopal responses. PAI-1 released from irradiated tumors also enters the circulation and triggers SFRP2^high CAF transdifferentiation at distant sites through the same pathway (47). PAI-1 additionally promotes angiogenesis, supports tumor survival, and upregulates PD-L1 (57). Because stroma-enriched tumors contain abundant pericytes and fibroblast progenitors, radiotherapy-induced PAI-1 elevation generates large numbers of SFRP2^high CAFs (47). SFRP2 overexpression remodels the extracellular matrix, disrupts vascular stability, and prevents immune infiltration. PAI-1 also activates broad immunosuppressive programs that contribute to immune tolerance (58). Myeloid-dominated tumors behave differently. ICD-released DAMPs are present, but local myeloid cells process them according to the surrounding microenvironmental context, a pattern we provisionally describe as myeloid reprogramming, though it has not yet been validated as a distinct clinical mechanism.

In these tumors, immunosuppressive signaling operates through several interconnected pathways, organized hierarchically with AREG as an upstream gating signal. Radiation-induced AREG engages EGFR on TAMs within hours of irradiation, reducing phagocytic capacity and suppressing antitumor immunity before downstream ICD signals fully accumulate (48); This AREG-conditioned myeloid state re-routes subsequent signals: extracellular ATP, which activates NLRP3 via P2X7 receptors and would otherwise drive robust T cell priming, instead preferentially recruits MDSCs and pro-tumorigenic TAMs under prolonged signaling in the AREG-preconditioned context (59); and sustained STING signaling, which acutely enhances DC maturation, instead upregulates PD-L1 and IDO under chronic DNA damage conditions, shifting the balance toward immune tolerance rather than sustaining DC maturation (54). Pathways that may favor immune activation include HMGB1 engaging TLR4 on dendritic cells to initiate maturation and T cell priming, though the outcome depends on HMGB1 oxidation state and local metabolic conditions; calreticulin binding CD91 to enable phagocytosis and cross-presentation, though high concentrations of PAI-1, TGF-β, and AREG can shift this toward tolerance (60, 61); and STING activation under low-dose radiotherapy, which generates IFN-β to support DC migration without triggering chronic suppression. AREG thus acts as a permissive switch, while ATP and type I IFN serve as amplifiers rather than independent initiators of myeloid suppression. Metabolic and oxidative conditions play an important role in determining which of these pathways prevails. High lactate, hypoxia, and reactive oxygen species favor suppressive myeloid polarization, while moderate metabolic stress can drive myeloid cells toward a more immunostimulatory phenotype. Rather than three co-equal suppressors, we propose a hierarchical organization in which AREG functions as an upstream gating signal.

This framework may help explain why abscopal responses are rare, particularly with radiotherapy alone. Increasing ICD signal strength is unlikely to be sufficient on its own. What may matter more is altering the conditions under which myeloid cells interpret those signals, though this remains to be tested clinically. The functional outcome of ICD depends on microenvironment-specific priority pathways: PAI-1/SFRP2 in stroma-enriched tumors, and the combined effects of AREG, HMGB1, and ATP signaling in myeloid-dominant tumors. Combination strategies should therefore be designed with the specific microenvironmental composition of individual tumors in mind, though the clinical feasibility of such tailored approaches has yet to be established.

### 2.3 Immune-desert microenvironments: angiogenesis-driven immune suppression

Immune-desert tumors lack sufficient T cells to mount ICD-dependent responses. CD8^+^ T cell densities often fall below 100 cells/mm², even in tumors with high mutational burden. This suggests that non-antigenic suppressive mechanisms play a dominant role in driving immune exclusion.

Two subtypes exist. “Cold” deserts lack immune recognition. MHC class I expression is reduced or absent, and the T cell-recruiting chemokines CXCL9, CXCL10, and CXCL11 are not produced. Without antigen presentation or chemotactic signals, adaptive immune responses do not initiate. These tumors generally do not respond to ICD-enhancing therapies alone (62). In cold deserts, restoring antigen presentation constitutes the essential therapeutic prerequisite before any ICD-based strategy can be effective. STING agonists have been shown to upregulate MHC class I surface expression on tumor cells in preclinical models (63), providing the immune visibility that ICD signals alone cannot confer when the antigen presentation machinery is absent. TLR agonists may further reconstitute the CXCL9/CXCL10 chemokine gradient required for dendritic cell recruitment and T cell priming. “Excluded” deserts retain immunogenic capacity. MHC-I is preserved, neoantigen loads are high, and tumor-specific T cells are detectable in circulation. The barrier is physical and molecular, not antigenic. Excessive VEGF signaling creates disorganized vessels that lack ICAM-1 and VCAM-1, the adhesion molecules T cells need to exit the bloodstream (64). Dense stromal matrices raise interstitial pressure and limit T cell penetration. FAS ligand on tumor endothelium induces apoptosis in CD8^+^ T cells as they attempt to infiltrate (65). Preclinical data support vascular normalization and stromal remodeling as the primary therapeutic entry point in excluded deserts, but this has not been confirmed in randomized clinical trials.

The treatment boundary between these two subtypes is therefore functionally defined: cold deserts require immune re-education, restoration of antigen presentation and chemokine signaling, as a prerequisite before ICD-derived signals can engage the adaptive immune system, whereas excluded deserts require physical barrier disruption to permit T cell entry. Conflating these two subtypes in trial design may account for some of the inconsistent outcomes observed with ICD-based strategies.

### 2.4 Mixed and transitional microenvironments: spatial and temporal dynamics

Most tumors are spatially heterogeneous and change over time in response to treatment, disease progression, and cellular interactions. Radiotherapy contributes to these transitions, and the resulting mixed microenvironmental states reflect underlying plasticity (66). Spatial transcriptomics data show that hypoxic, poorly perfused tumor cores develop immune-desert features, while peripheral regions near functional vasculature tend to be immune-active or stroma-enriched (67) (68). Lymph node metastases are often densely T cell-infiltrated, while visceral metastases tend to become suppressive and dominated by myeloid cells or stroma. Radiotherapy reorganizes the microenvironment over days to weeks. A well-documented pattern in preclinical models is a shift from immune-desert to myeloid-enriched states. Radiation damages blood vessels, which triggers the release of VEGF and CXCL12 and recruits Ly6C^+^ monocytes and neutrophils. As hypoxia deepens, these cells differentiate into suppressive macrophages and MDSCs. In preclinical models, this suppressive compartment peaks around days 3–7 after irradiation (69). Preclinical data suggest a potential therapeutic window at 48–72 hours post-irradiation, before suppressive myeloid programs are established. During this window, AREG neutralization or CSF1R blockade might redirect myeloid differentiation toward less immunosuppressive phenotypes. This idea is based on preclinical evidence and has not been tested clinically. A second pattern involves the stroma. Radiation activates CAFs, which secrete CCL2 and CXCL12 to recruit myeloid cells and upregulate PAI-1 to tighten stromal barriers. The combined effect of activated CAFs and recruited myeloid cells creates a state of “stroma-myeloid cooperative suppression” that is highly resistant to treatment (70, 71). Preclinical data suggest that stromal expansion occurs mainly in the first week after radiotherapy, while myeloid suppression builds over subsequent weeks. Based on these preclinical observations, combining stromal barrier disruption (such as PAI-1 or SFRP2 inhibition) with myeloid targeting (such as AREG or CD47 blockade), timed around radiotherapy, has been proposed as a potential strategy worth investigating.

## 3 Prospects: toward a “precision barrier-breaking” strategy for reliable ICD induction

This section presents a hypothesis-driven framework based on preclinical and translational evidence. The strategies described carry mechanistic rationale but require prospective clinical validation before clinical application.

Two principles underpin this framework. First, ICD must be strong and sustained enough to release adequate tumor antigens and DAMPs to initiate systemic antitumor immunity; weak ICD cannot be compensated downstream (72, 73). Second, metastatic lesions differ in suppressive architecture. Some are stroma- and CAF-enriched, others are myeloid-dominated, and others show T cell exhaustion or exclusion. Identifying the dominant resistance mechanism in each lesion and selecting combinations accordingly is rational, though clinical validation is needed (74, 75).

Radiotherapy, oncolytic viruses, STING agonists, and certain chemotherapeutics act on shared ICD pathways: the cGAS-STING-IFN axis, ER stress-driven calreticulin externalization, the ATP-P2RX7-NLRP3 inflammasome cascade, and HMGB1-TLR4/RAGE signaling. Conventional low-dose radiotherapy produces limited ICD signals. Combining STING agonists with radiotherapy increases DAMP release and antigen presentation in preclinical models, and early clinical data suggest systemic responses may be promoted, though optimal delivery remains under investigation (76, 77). Anthracyclines and oxaliplatin have characterized DAMP-inducing properties and may amplify DAMP release when combined with radiotherapy (61). Oncolytic viruses lyse tumor cells, create inflammation, and drive antigen release; combining them with radiotherapy shows promise in preclinical and early-phase studies (78, 79).

We propose a three-step framework: (1) multi-lesion sampling and microenvironmental subtyping, (2) lesion-specific combination regimens based on those findings, and (3) biomarker and imaging monitoring to guide adjustments. Subtyping uses histopathology and multi-site biopsies with spatial transcriptomics, multiplex immunofluorescence, and imaging mass cytometry to identify SFRP2-high CAFs, quantify myeloid infiltration, and map T cell exclusion patterns; such classifications correlate with immunotherapy responses in preclinical and translational studies (74, 80). When biopsies are not feasible, radiomic features, PET imaging, and ctDNA may serve as alternatives, though their predictive value for abscopal outcomes needs validation (81, 82).

**Figure 4:**
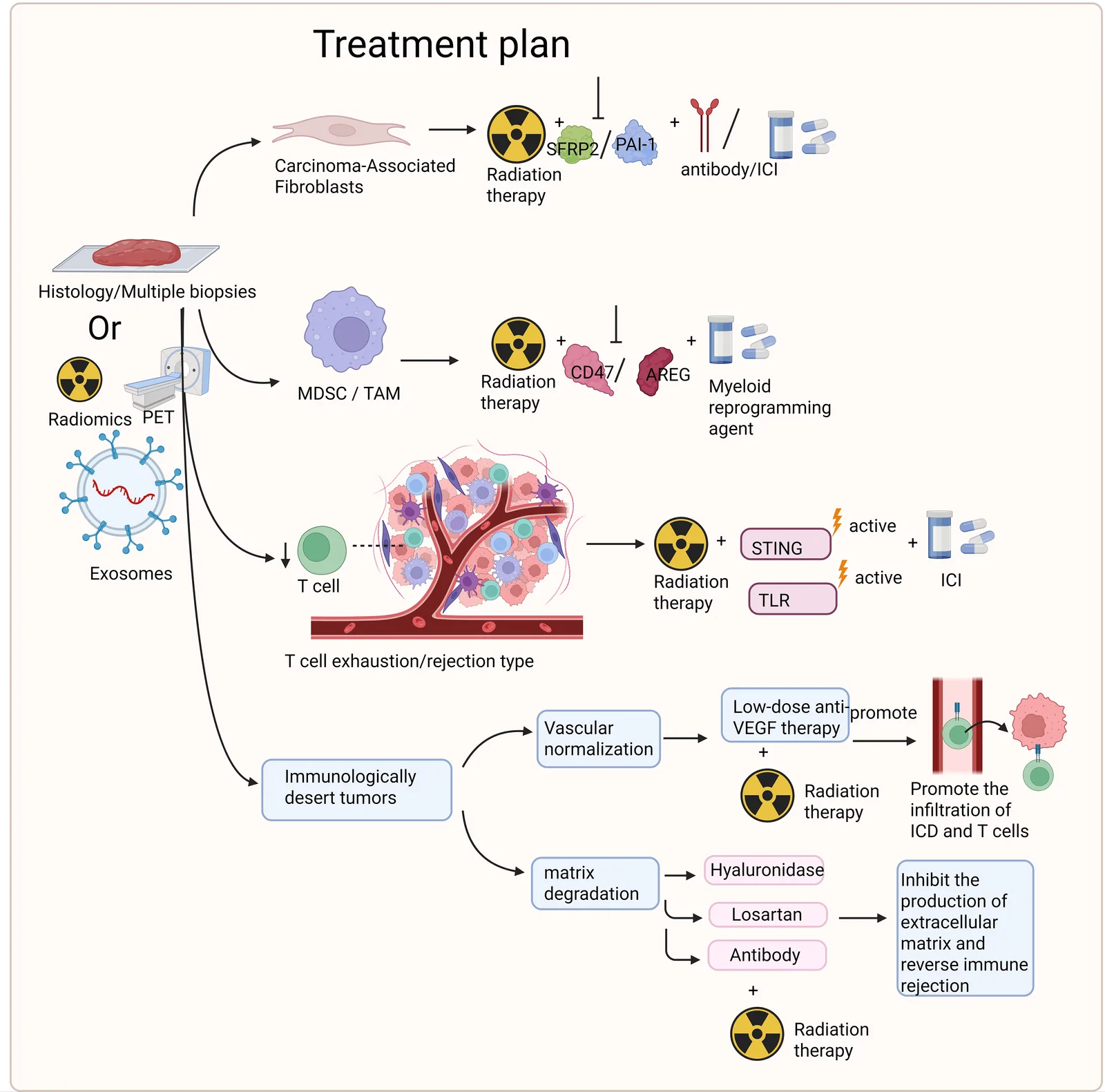
Subtype-guided investigational radio-immunotherapy framework. TME subtyping is performed via histology, radiomics, PET, or exosomes. Four lesion-specific strategies are proposed: (1) stroma-enriched: RT + PAI-1/SFRP2 inhibition + ICI; (2) myeloid-enriched (MDSC/TAM): RT + CD47/AREG blockade + myeloid reprogramming agents; (3) T cell exhaustion/exclusion: RT + STING/TLR activation + ICI; (4) immune-desert: vascular normalization (low-dose anti-VEGF + RT) and matrix degradation (hyaluronidase/losartan/antibody + RT) to promote ICD and T cell infiltration. Transitional states may require combined strategies.

Lesion-specific regimens follow from this subtyping. In stroma- and CAF-enriched lesions, CAF-secreted factors promote radiotherapy resistance and reduce T cell infiltration in preclinical models; SFRP2 correlates with stromal drug resistance, and PAI-1 is linked to PD-L1 upregulation (47, 83, 84). The proposed strategy combines moderate-dose radiotherapy locally with SFRP2-neutralizing antibodies or PAI-1 inhibitors, together with systemic PD-1/PD-L1 blockade and CAF-phenotype-selected TGF-β inhibition, jointly targeting the stromal barrier and CAF-driven PD-L1 upregulation. In myeloid-enriched lesions, high TAM and MDSC infiltration suppresses immunity through phagocytosis inhibition, and AREG upregulates CD47 to drive post-radiation immunosuppression in preclinical models (48, 85, 86). The proposed strategy pairs radiotherapy locally with oncolytic viruses or STING agonists, combined systemically with anti-CD47 therapy or SIRPα antagonists, AREG inhibitors, and optional CSF1R inhibitors (**Figure 4**).

T cell infiltration is required for abscopal effects, but in cold tumors with low mutational burden or defective antigen presentation, ICD-induced antigen release may fail to recruit T cells. In preclinical models, combining radiotherapy with STING agonists, TLR agonists, or oncolytic viruses and checkpoint inhibitors has converted cold tumors into more immunologically active ones; clinical translation is under investigation (36, 78). In immune-inflamed lesions, low-dose radiotherapy may refresh antigen presentation without inducing immunosuppression, with systemic checkpoint blockade or intratumoral co-stimulatory molecules.

Immune-desert tumors present a distinct challenge: when T cells and DCs are nearly absent, danger signals have no responding cells. A preclinical staged approach proposes restoring vascular access first, then amplifying inflammation. Low-dose anti-VEGF normalizes vasculature at days 3 to 7 (87); PEGPH20 degrades hyaluronan to lower interstitial pressure (88); losartan or direct TGF-β neutralization reduces collagen deposition. Once barriers are reduced, STING agonists or oncolytic viruses may support T cell entry via CXCL10 (89).

Clinical experience with each proposed strategy warrants caution. Regarding stromal targeting, CAF depletion accelerated tumor progression in preclinical models by removing tumor-restraining stromal components (90), confirming that microenvironmental remodeling rather than ablation is the appropriate goal. HALO-301 tested PEGPH20 in combination with chemotherapy in prospectively selected hyaluronan-high pancreatic cancer patients and still failed to improve overall survival despite biomarker-enriched enrollment (91), indicating that single-pathway stromal intervention is insufficient and that compensatory suppressive mechanisms likely operate in parallel. Regarding STING activation, intratumoral ADU-S100 produced limited objective responses in phase I trials despite measurable systemic immune activation (92), consistent with the principle that STING stimulation alone cannot overcome intact suppressive barriers. Magrolimab was discontinued across hematologic and solid tumor programs due to safety and efficacy concerns, reinforcing that CD47 blockade requires patient selection based on myeloid enrichment and AREG/CD47 co-expression rather than broad application (93). These clinical failures collectively indicate that microenvironment-matched, multi-axis combination design, rather than single-target intervention in unselected populations, is a prerequisite for reproducible clinical efficacy.

Because the TME changes rapidly after radiotherapy, serial monitoring is needed at three time points: (1) baseline multi-region biopsies or spatial transcriptomics to map immune architecture; (2) days 3 to 7, liquid biopsy markers and DW-MRI to capture early remodeling; (3) weeks 3 to 4, metabolic changes in non-irradiated lesions and ctDNA kinetics as early abscopal indicators (**Table 2**).

**Table 2:** Preclinical basis and clinical implications of proposed assessment checkpoints for ICD-driven immune responses.

Phase	Major Biological Events	Key Therapeutic Interventions	Contraindications/Avoidances	Monitoring Markers
0-6h	CRT exposure, ATP release, ROS burst	STING agonist (concurrent), OV (immediate/delayed)	High-dose antioxidants, corticosteroids	Tumor site temperature (inflammation)
6-24h	cGAS-STING activation, IFN-I peak	TLR agonists (12-18h), initial anti-PD-L1 optional	High-dose antioxidants, corticosteroids	Circulating IFN-β, CXCL10
1-3d	HMGB1 peak, myeloid recruitment, PAI-1/AREG surge	Anti-PAI-1 (start <24h), Anti-AREG (6-48h), metabolic modulators (pre-treatment)	Delayed barrier blockade (>72h less effective)	Circulating PAI-1, AREG, MDSC frequency
3-7d	DC migration, T cell priming, clonal expansion	Initial PD-1/PD-L1 (5-7d), vaccine boost (7-14d)	Premature barrier blockade cessation	Lymph node size, peripheral tumor-specific T cells
>7d	T cell infiltration abscopal, memory formation	Maintain checkpoint inhibition, adaptive adjustments	Premature regimen change for pseudoprogression	ctDNA, PET-CT, abscopal lesion size

Based on preclinical evidence (94), we propose a two-step sequence: a strong local ICD stimulus via radiotherapy with or without oncolytic viruses or intratumoral STING agonists, followed within 24 to 72 hours by systemic therapies to suppress compensatory immunosuppression. Monitoring uses cfDNA, exosomal DAMPs, immune profiling, and serial liquid biopsies (81, 82). Optimal scheduling requires prospective testing.

We propose randomized trials in patients with metastatic solid tumors bearing two or more measurable lesions, comparing systemic therapy alone versus systemic therapy plus lesion-targeted radiotherapy, with non-irradiated lesion response rate as the primary abscopal endpoint. A no-radiation comparator arm is essential: as demonstrated in the PEMBRO-RT trial design (2), without such a control, regression at non-irradiated sites cannot be confidently attributed to radiotherapy rather than to the systemic agent acting independently (5). Prior to randomization, each patient undergoes multi-lesion microenvironmental profiling to identify the dominant suppressive subtype; the index lesion is selected for irradiation based on this profile, and the systemic agent is matched to the corresponding priority pathway.

A key implementation challenge is that systemic agents inherently affect all metastatic deposits, not only the irradiated index lesion, confounding attribution of abscopal responses. Intratumoral or locoregional delivery of subtype-matched agents offers a potential solution: for stroma-enriched lesions, intratumoral PAI-1 inhibitors or SFRP2-neutralizing antibodies concentrate barrier disruption at the irradiated site; for myeloid-enriched lesions, intratumoral STING agonists or oncolytic viruses provide localized myeloid reprogramming, in both cases combined with systemic checkpoint blockade (76–78, 95). This approach limits confounding of non-irradiated lesion responses by isolating the dominant suppressive axis intervention to the irradiated site. Mechanistic co-endpoints should include on-treatment biopsies at 48–72 hours post-irradiation, serial ctDNA kinetics, exosomal DAMP profiling, and TIL composition at both irradiated and non-irradiated sites (74, 96, 97). Separation of radiotherapy-specific from systemic drug effects should be pre-specified as a secondary analysis.

Abscopal effects are not entirely explained by ICD. In preclinical models, combining radiotherapy with CD47 inhibition produced abscopal effects even without T cells, through macrophage migration (98). Because most observed abscopal effects correlate with ICD, this review focuses on that mechanism, while noting that non-ICD pathways also deserve investigation.

## 4 Conclusion

The abscopal effect offers a theoretical basis for achieving systemic antitumor responses through local radiotherapy, yet it remains clinically rare. ICD has been proposed as a key driver, but whether it promotes systemic immunity or shifts toward tolerance likely depends on the tumor’s pre-existing suppressive architecture. We identify two distinct patterns: stroma-enriched tumors erect broad inhibitory barriers blocking immune activation at multiple levels, while myeloid-enriched tumors selectively reinterpret rather than globally silence ICD signals, and these two architectures may ultimately determine whether ICD drives effective immunity or is neutralized by competing suppressive cues.

On this basis, we propose a “precision barrier-breaking” strategy that functionally subtypes the TME and targets its dominant suppressive mechanism, extending existing classifications by asking not only what microenvironment is present, but which inhibitory pathway is most active and when intervention is most feasible. The goal is not to amplify ICD per se, but to time interventions so that ICD signals arrive during windows of microenvironmental receptivity. This framework remains hypothesis-generating, and prospective clinical validation is required before it can guide practice; nevertheless, it may offer a mechanistic rationale for moving the abscopal effect from a rare observation toward a more reproducible outcome.
